# Ventriculoscopy combined with Ommaya reservoir implantation for treatment of hydrocephalus with atypical neuroimaging features of neurocysticercosis: A case report

**DOI:** 10.1097/MD.0000000000042320

**Published:** 2025-05-02

**Authors:** Qiang Chen, Qiang Shao, Lang Chen

**Affiliations:** a Department of Neurosurgery, General Hospital of Yangtze River Shipping, Wuhan Brain Hospital, Wuhan, Hubei Province, China.

**Keywords:** cysticercosis, hydrocephalus, ommaya reservoir, ventriculoscopy

## Abstract

**Rationale:**

Cysticercosis-induced hydrocephalus presents diagnostic and therapeutic challenges owing to its heterogeneous clinical manifestations. Acute hydrocephalus secondary to parasitic infections of the central nervous system (CNS) requires tailored surgical interventions to address impaired cerebrospinal fluid (CSF) dynamics and intracranial hypertension.

**Patient concerns:**

A 27-year-old male reported intermittent dizziness and headaches persisting for 3 years and a recent onset of vomiting over the past month.

**Diagnoses:**

Neuroimaging demonstrated ventricular enlargement, thickened basal membranes, and elevated intracranial pressure, without classical cysticercosis imaging markers. Serological and CSF enzyme-linked immunosorbent assay (ELISA) tests confirmed the presence of antibodies against cysticercosis. The patient was diagnosed with acute exacerbation of chronic hydrocephalus secondary to a CNS cysticercosis infection complicated by extensive ependymitis and CSF absorption dysfunction.

**Interventions:**

Ventriculoscopy identified impaired CSF circulation caused by ependymitis, precluding conventional ventriculoperitoneal (V-P) shunts or endoscopic third ventriculostomy. An Ommaya reservoir was implanted to regulate intracranial pressure, which was supplemented by antiparasitic therapy with praziquantel.

**Outcomes:**

Postoperative follow-up confirmed resolution of hydrocephalus and complete alleviation of neurological symptoms, with no complications observed.

**Lessons:**

This case highlights ventriculoscopy-guided Ommaya reservoir implantation as a safe and effective alternative for managing parasitic infection-induced hydrocephalus when standard surgical options are contraindicated. This strategy addresses both CSF dynamics and infection and provides a reference for managing atypical neurocysticercosis cases.

## 1. Introduction

Neurocysticercosis (NCC), caused by the larval stage of the pork tapeworm (Taenia solium), is a prevalent parasitic infection that frequently affects the central nervous system (CNS). This infection can manifest with a spectrum of neurological symptoms, including seizures, headache, cognitive decline, and hydrocephalus in severe cases.^[1,2]^ Accurate diagnosis of cysticercosis-induced hydrocephalus is essential for its effective management. Identifying the source of infection and characterizing brain lesions are crucial steps in determining the appropriate medical and surgical interventions. The location, number, and size of the cysticerci, and the associated inflammatory response significantly influence treatment choices. The diagnosis of NCC relies primarily on neuroimaging studies, which are supported by immunological detection in serum and cerebrospinal fluid (CSF) and histopathological examination of resected lesions when feasible. Epidemiological evidence also supports the diagnosis.^[3]^ Next-generation sequencing (NGS) of CSF has emerged as a promising diagnostic tool for NCC, offering high sensitivity and specificity for pathogen detection.^[4–7]^ However, despite advances in these diagnostic tools, challenges persist in treating patients with suspected NCC, owing to heterogeneous clinical manifestations.^[2,3,8]^

The choice of surgical procedure depends on the type and severity of the hydrocephalus. Ventriculoperitoneal (V-P) shunts are the most common surgical treatment for hydrocephalus. Endoscopic third ventriculostomy (ETV) is a minimally invasive procedure that creates a new pathway for cerebrospinal fluid (CSF) flow that bypasses the obstruction. Surgical removal of the cysticerci may be necessary to relieve mechanical obstruction and reduce inflammation. Nevertheless, in cases where the neuroimaging features are atypical and there is an infection-associated severe inflammatory response, these surgical procedures are not suitable.^[2,3,9]^

Ommaya reservoir implantation is often used to reduce intracranial pressure in patients with acute hydrocephalus by allowing intermittent aspiration of CSF and drug delivery into the CSF.^[10,11]^ However, its clinical utility in NCC treatment remains largely unexplored. In this study, we report the pioneering use of ventriculoscopy combined with Ommaya reservoir implantation to manage acute hydrocephalus caused by NCC. Additionally, we reviewed 8 previously reported cases of cysticercosis-induced hydrocephalus from the literature to analyze the clinical complexities encountered during diagnosis and treatment.

## 2. Case report

A 27-year-old male patient was admitted to the hospital with a 3-year history of intermittent dizziness, headaches, and a recent onset of vomiting over the past month. He denied any fever, limb convulsions, or altered consciousness. His past medical history revealed occupational exposure as a chef, with frequent handling of raw and cooked food. Notably, between 2015 and 2016, he had experienced multiple episodes of passing small worms in his stool, which resolved after self-administering anthelmintic medication. Upon admission, the patient was alert and oriented. His pupils were equal and reactive to light and measured 3 mm in diameter. The neck was supple, with no signs of meningeal irritation. No subcutaneous nodules were observed. Motor examination revealed normal muscle strength (grade 5) in all 4 limbs.

**Figure 1.**
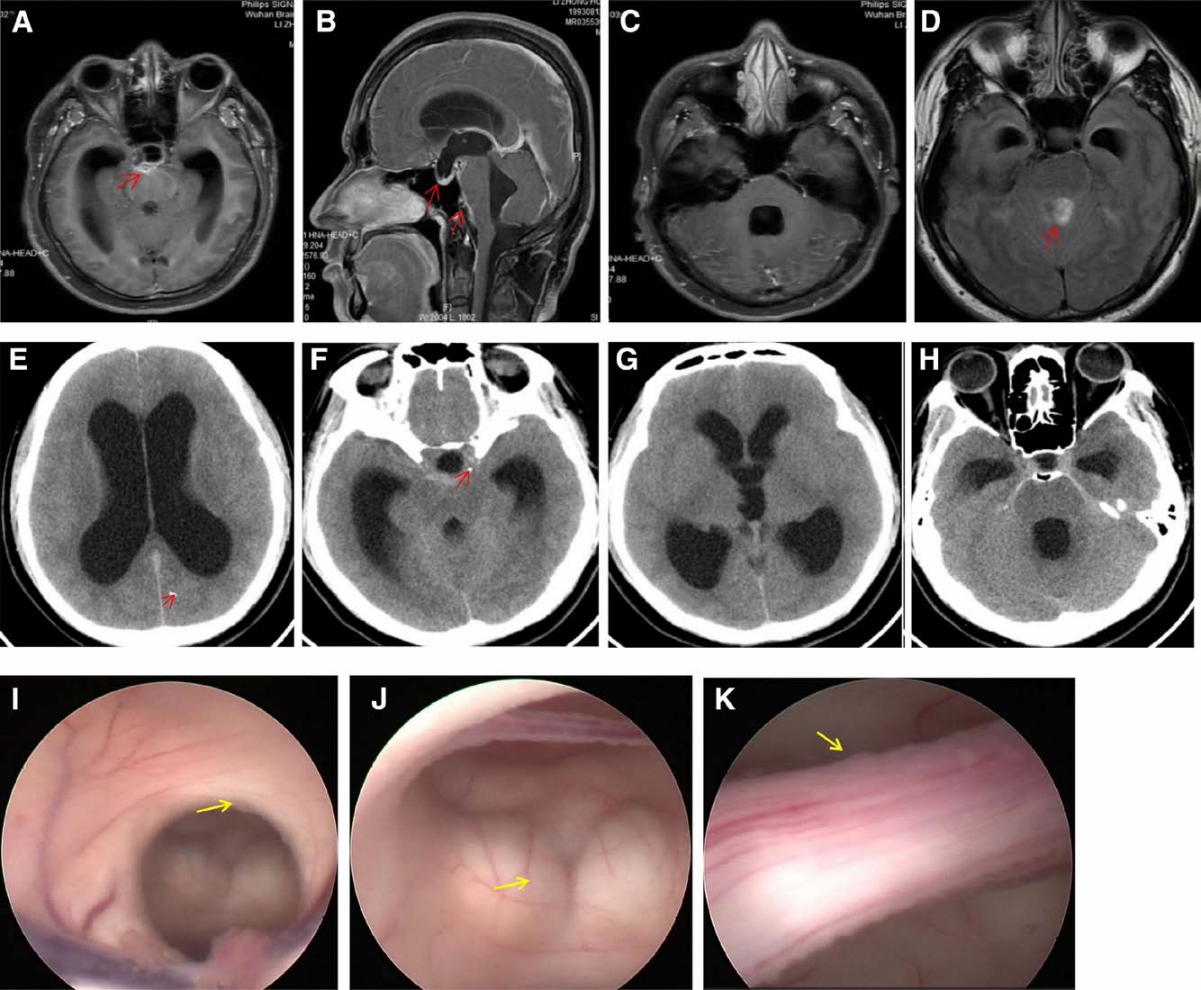
Preoperative neuroimaging and intraoperative ventriculoscope exploration. On admission, MRI showed: (A) inflammation and adhesion of the basal cisterns with enlarged lateral ventricles. (B) Enlarged third ventricle with downward herniation and basal cistern inflammation. (C) Enlarged fourth ventricle. (D) Heterogeneous signals within ventricles (FLAIR imaging). Three weeks later, head CT revealed (E) calcified lesions in the left occipital lobe, enlarged lateral ventricles, and shallow sulci. (F) Calcified lesions in the parasellar region. (G) Enlarged lateral and third ventricles. (H) Enlarged temporal horns and the fourth ventricle. Intraoperative ventriculoscopy showed (I) white, fine granular, and membranous proliferations covering the ventricular edges. (J) The mammillary bodies are covered by inflammatory proliferations obscuring the basilar artery. (K) Enlarged interpeduncular fossa with granular proliferation. CT = computed tomography, FLAIR = fluid attenuated inversion recovery, MRI = magnetic resonance imaging.

Upon admission, the patient underwent magnetic resonance imaging (MRI) with both plain and contrast-enhanced sequences. The MRI revealed marked enlargement of the lateral, third, and fourth ventricles, with downward displacement of the third ventricle and thickening of the basal cisterns (Fig. 1A–D). Concurrently, CSF analysis revealed elevated white blood cell counts and protein levels, along with slightly decreased glucose levels (white blood cells: 98 to 159 × 10^6^/L, protein: 121.8 to 172 mg/dL, glucose: 2.34 to 2.72 mmol/L, chloride: 118.9 to 126.2 mmol/L).

Extensive pathogen testing was performed, including CSF pathogen smears and cultures, which were negative for viruses, tuberculosis, cryptococcus, and other bacteria. Fecal microscopy of the parasites was also negative. NGS of the CSF did not detect fungi, viruses, parasites, or M. tuberculosis. However, in-house dot-enzyme-linked immunosorbent assay (dot-ELISA) testing of both the serum and CSF revealed positive results for antibodies against T. solium, E. granulosus and S. mansoni. Three weeks after admission, the patient experienced significant deterioration in symptoms, characterized by worsening headaches and frequent vomiting. Follow-up head computed tomography (CT) demonstrated increased cerebral edema, with shallowing of the sulci and gyri, indicative of elevated intracranial pressure (Fig. 1E–H).

**Figure 2.**
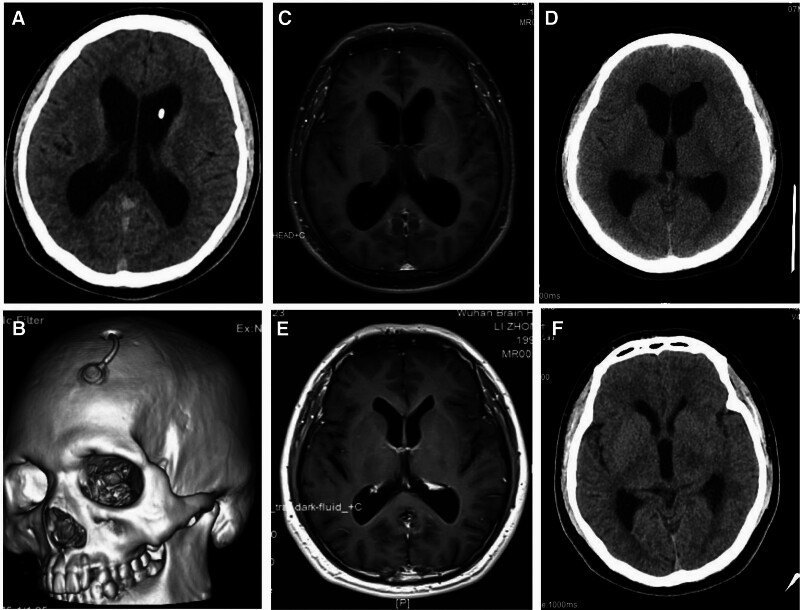
Postoperative CT scan. Head CT scan after surgery showed that (A and B) the drainage tube and Ommaya reservoir were well positioned. Head CT scan for recovery status at 1.5 (C), 5 (D), 11 (E), and 14 mo (F) postoperatively. CT = computed tomography.

Ventriculoscopy was planned to further explore intraventricular lesions. Specifically, a hard STORZ-lotte ventriculoscope was inserted at the Kocher point. Under direct visualization, the ventricles were found to be extensively covered with a thin, white, granular, and membranous proliferative material. The floor of the third ventricle, anterior to the mammillary bodies, was significantly sunken, and the local arachnoid membrane was white and markedly thickened, obscuring the view of the basilar artery and other neural structures. Exploration within the third ventricle revealed that the interpeduncular fossa was coarse and covered with granular proliferative material. During endoscopic irrigation, proliferations tightly adherent to underlying structures were found, complicating biopsy due to bleeding risk. Additionally, the structure at the Liliequist membrane, located at the floor of the third ventricle, appeared indistinct, likely due to old inflammatory adhesions. To avoid damaging the basilar artery, biopsy was not performed. Further exploration toward the posterior aspect revealed a patent cerebral aqueduct with a diameter of approximately 5 mm (Fig. 1I–K). CSF drainage was subsequently performed and an Ommaya reservoir was implanted at Kocher point. The ventricular end of the catheter was inserted and connected to the Ommaya reservoir, which was secured beneath the scalp. Postoperative follow-up head CT confirmed that the drainage tube and Ommaya reservoir were well positioned (Figure 2A and B).

The patient underwent antiparasitic treatment with praziquantel following surgery, supplemented with intermittent and direct CSF drainage via the Ommaya reservoir. The treatment was administered in multiple courses over a total duration of 5 months (Table 1). One and a half months after treatment initiation, the patient’s headache symptoms resolved completely. Postoperatively, the patient did not experience any symptoms of increased intracranial pressure, such as headache, nausea, or vomiting.

**Table 1:** Postoperative treatment.

Course	Medication	Dosage	Frequency	Duration	Additional procedures
1	PRA	0.4 g	Oral, 3 times/d	10 d	Continuous drainage via Ommaya reservoir, 200 mL/d for 2 wk
Interval	—	—	—	15 d	—
2	PRA	0.4 g	Oral, 3 times/d	10 d	Puncture via Ommaya reservoir, 50 mL/d for 10 d
Interval	—	—	—	15 d	—
3	PRA	0.4 g	Oral, 3 times/d	10 d	Puncture via Ommaya reservoir, 50 mL every 2 d
Interval	—	—	—	15 d	—
4	PRA	0.4 g	Oral, 3 times/d	10 d	Puncture via Ommaya reservoir, 50 mL every 2 d
Interval	—	—	—	15 d	—
5	PRA	0.4 g	Oral, 3 times/d	10 d	—
Interval	—	—	—	15 d	—
Duration	—	—	—	—	Continue until 5 mo of treatment

PRA = praziquantel.

During follow-up, biochemical analysis of the CSF revealed a progressive normalization of the cell count, protein levels, and glucose concentrations (Table 2). CT imaging revealed controlled hydrocephalus at 1.5 and 5 months postoperatively, and a marked reduction and stabilization of ventricular morphology at 11 and 14 months postoperatively (Fig. 2C–F). After a 2-year follow-up period, the patient’s hydrocephalus was fully stabilized and resolved, thereby eliminating the need for ventriculoperitoneal shunt surgery.

**Table 2:** Postoperative features of laboratory tests of CSF.

Time point	WBC (10^6^/L)	Protein (mg/dL)	Glucose (mmol/L)	Chloride (mmol/L)
Day 1 post-surgery	112	85	2.22	117.3
1.5 mo post-surgery	94	123	2.52	106.4
2.5 mo post-surgery	35	35	2.85	125.1
5 mo post-surgery	6	25	3.15	126.7

CSF = cerebrospinal fluid, WBC = white blood cells.

Normal reference range: WBC: 0 to 8 × 10^6^/L; protein: 15 to 45 mg/dL; glucose: 2.5 to 4.5 mmol/L; chloride: 120 to 132 mmol/L.

## 3. Discussion

Cysticercosis is one of the most common parasitic infections, with 60% to 90% of patients developing CNS involvement. The eggs of the pork tapeworm are typically ingested orally and hatch in the duodenum, where the primary larvae (hexacanth) emerge from the cysts, penetrate the intestinal wall, enter the bloodstream, and migrate to the brain, where they develop into secondary larvae (cysticerci) and become established.^[12]^ Depending on the affected anatomical site, the disease is commonly classified into parenchymal, ventricular, or subarachnoid forms. Mechanical obstruction and inflammation are the key pathophysiological mechanisms underlying hydrocephalus in NCC. Intraventricular cysticerci can directly obstruct CSF circulation, leading to hydrocephalus.^[13]^ Infections involving the ventricles or subarachnoid space often result in arachnoiditis and ependymitis, disrupting CSF dynamics and causing hydrocephalus. Hydrocephalus associated with NCC leads to increased intracranial pressure (ICP) and cerebral dysfunction, significantly increasing mortality risk.^[14,15]^

Typical MRI findings of parenchymal neurocysticercosis include cystic lesions with distinct nodules, which are relatively straightforward to diagnose. However, some lesions may be too small to be detected by MRI, presenting only as hydrocephalus or enhancement of the basal meninges, particularly when the lesions are located within the ventricles or subarachnoid space.^[16]^ In the present case, imaging studies revealed only enhancement of the basal membranes, with no detectable cysticercal nodules. The diagnosis was established based on a comprehensive evaluation of the following findings: Neuroimaging revealed enlarged ventricles, thickening of the basal membranes, and increased intracranial pressure. Repeated lumbar punctures showed elevated cell counts and protein levels in the CSF, with mildly decreased glucose levels, suggesting an infectious etiology. Epidemiological evidence of potential occupational contact with *Taenia solium* infection. Pathogen testing indicated positive results for antibodies against cysticercosis, echinococcosis, and sparganosis in the serum and CSF ELISA tests. Ventriculoscopy revealed signs of cysticercosis infection, with slightly turbid CSF and the ventricular walls covered with fine granular and membranous excrescences. Antiparasitic treatment resulted in improved clinical symptoms, neuroimaging, and CSF findings.

Through a literature review, we identified 8 relevant cases of NCC patients with hydrocephalus who lacked typical neuroimaging features (Table 3).^[4,5,17]^ In patients 1 to 4, cerebral MRI showed an expanded ventricular system and hydrocephalus, with an initial diagnosis of tuberculous meningitis. Patients 5 to 7 were diagnosed with intraventricular NCC, basal NCC, and combined basal subarachnoid and parenchymal NCC, respectively. For patients 1 to 7, a diagnosis of probable NCC was not made until positive CSF NGS and immunological tests were performed. In patient 8, MRI revealed a nonspecific lesion, and pathological analysis of the resected lesion identified the parasite as *Taenia solium*. NGS plays a crucial role in the process of determining the source of infection in patients 1 to 7, which is consistent with previous reports regarding its outstanding specificity and sensitivity in pathogen detection, especially in patients with atypical features in NCC identification.^[6,7]^ However, in our patient, CSF NGS yielded negative results, despite positive serum and CSF ELISA results. This discrepancy may be attributed to the timing of sample collection and prior treatment, which could have influenced the pathogen DNA load in the CSF. Alternatively, genetic sequence variation in pathogens may also have contributed to the negative NGS findings.^[6,18,19]^ In contrast, the results of the immunological antibody tests depend on the presence and extent of the inflammatory response, which is consistent with the extensive ventricular inflammation observed in our patient. This also suggests that negative NGS results alone may not rule out NCC, and that multiple diagnostic approaches should be considered. Our study also highlights the importance of considering the epidemiological context. Notably, in our case, ELISA for antibodies against T. solium, E. granulosus and S. mansoni yielded positive results. This suggests the potential for cross-reactivity and false positives in these assays, a phenomenon that has been documented in other studies.^[20,21]^

**Table 3:** Literature review of hydrocephalus with atypical NCC neuroimaging features.

CaseNo.	Age	Clinical manifestations	Diagnosis				Treatment
			Neuroimaging	NGS	Immunological test (antibody)	Other tests	
1	44	Seizure, headache, transient LOC, cognitive decline	MRI showed an expanded ventricular system and hydrocephalus	CSF (+)	Serum and CSF (+)	None	ABZ, DXM, ORI
2	50	Headache, nausea, vomiting	MRI showed an expanded ventricular system and hydrocephalus	CSF (+)	Serum and CSF (+)	None	ABZ, DXM
3	41	Recurrent fever, headache, nausea, vomiting	MRI showed an expanded ventricular system and hydrocephalus	CSF (+)	Serum and CSF (+)	None	PRA, DXM
4	47	Headache, nausea, vomiting	MRI showed an expanded ventricular system and hydrocephalus	CSF (+)	Serum and CSF (+)	None	PRA, DXM
5	58	Headache, visual impairment, transient LOC, cognitive decline	MRI showed hydrocephalus and enhanced lesion posterior to the medulla	CSF (+)	Serum and CSF (+)	None	ABZ, DXM
6	31	Visual impairment	MRI showed hydrocephalus and multiple cystic lesions in the suprasellar cistern	CSF (+)	Serum and CSF (+)	None	ABZ, DXM, ETV
7	53	Seizure, headache, visual impairment, cognitive decline	CT showed scattered parenchymal calcified lesions; MRI showed hydrocephalus, enhancement of the basal meninges, and multiple cystic lesions	CSF (+)	Serum and CSF (+)	None	ABZ, DXM, ETV
8	23	Headache, nausea, hyper-reflexic	CT show significant dilation of the lateral ventricles; MRI show hydrocephalus and nonspecific lesion.	None	None	Resected lesion (+)	Lesion resection, V-P shunt, ABZ

ABZ = albendazole, CSF = cerebrospinal fluid, CT = computed tomography, DXM = dexamethasone, ETV = endoscopic third ventriculostomy, LOC = loss of consciousness, MRI = magnetic resonance imaging, NCC = neurocysticercosis, NGS = next-generation sequencing, ORI = Ommaya reservoir implantation, PRA = praziquantel, V-P = ventriculoperitoneal.

Age means the age at definitive diagnosis; + positive; − negative. References: (case 1–4),^[5]^ (case 5–7),^[4]^ (case 8).^[17]^

Traditional treatments for hydrocephalus include V-P shunt surgery, ETV, and cysticercus removal. V-P shunt surgery can rapidly alleviate hydrocephalus. However, owing to persistent inflammation in the CSF, up to 44.4% of patients experience shunt failure, primarily within the first year (66.7%).^[9]^ ETV has been reported to result in significant symptom improvement in 70% to 95% of cases, with 0% to 30% of cases requiring no further surgery.^[22]^ However, in patients with severe inflammatory reactions, ETV may fail owing to stoma closure.^[23]^ In our review of previously reported cases, the patients in cases 6 and 7 underwent ETV due to severe hydrocephalus, whereas the patient in case 8 underwent endoscopic ventriculoscopy with partial excision of the lesion followed by implantation of a V-P shunt. However, the shunt was removed 6 weeks postoperatively because of a distal catheter infection. In contrast, in our patient, the cysticerci were not clearly localized, and the patient had extensive basal adhesions due to infection. These factors make the patient unsuitable for any of the aforementioned surgical approaches.

The Ommaya Reservoir is an intraventricular implantation system that offers several advantages, including the ability to perform repeated punctures, simple operation, and long-term placement. It was connected to an intraventricular drainage tube via a subcutaneous reservoir. By puncturing the subcutaneous reservoir, the CSF can be aspirated or drained to remove inflammatory mediators, alleviate hydrocephalus, and facilitate intraventricular drug administration. The Ommaya reservoir has widespread applications in the treatment of brain metastases, CNS infections, intraventricular hemorrhage in infants, and intracranial cystic tumors.^[10,24]^ However, its clinical utility in the treatment of NCC-associated hydrocephalus remains unclear. In this study, we explored the use of an Ommaya reservoir in the patient with NCC-associated hydrocephalus. An Ommaya reservoir was implanted into the lateral ventricle to complement the antiparasitic treatment. Through external drainage and intermittent aspiration of CSF via the Ommaya reservoir, we effectively reduced the amount of inflammatory substances in the CSF and alleviated the inflammatory response caused by the cysticercosis infection. In addition, ICP elevation associated with acute hydrocephalus, as well as that occurring during antiparasitic treatment, were effectively managed. This approach successfully controlled both the infection and hydrocephalus, thereby eliminating the need for further shunt surgery.

However, this study has several limitations. First, a definitive histological diagnosis was not achieved. Second, the absence of manual eosinophil counts in the CSF limits the ability to provide strong evidence for a diagnosis of CNS parasitic infection and impairs the evaluation of treatment efficacy. Third, ventriculoscopy requires a high level of surgical skill from the operator and carries potential risks such as intraventricular hemorrhage and brain tissue damage. Therefore, systematic professional training is necessary.^[25]^ Additionally, the implantation of an Ommaya reservoir can be complicated by scalp and intracranial infections due to repeated punctures, shunt blockage, subdural hematoma or effusion, and intraventricular migration of cysticerci.^[23,26]^ Further multicenter clinical studies are needed to explore the clinical utility of ventriculoscopy and Ommaya reservoir implantation for the treatment of acute hydrocephalus caused by NCC.

In conclusion, our study demonstrates the safety and effectiveness of combining ventriculoscopy with Ommaya reservoir implantation for treating hydrocephalus associated with NCC. This integrated approach leverages the benefits of minimally invasive surgery and the high efficiency of local treatment, offering a novel therapeutic option for patients with atypical NCC-induced acute hydrocephalus. However, our findings were based on a limited number of cases, and further studies are needed to fully validate the general applicability and long-term efficacy of this therapeutic strategy.

## Author contributions

**Conceptualization:** Qiang Shao.

**Data curation:** Lang Chen.

**Funding acquisition:** Lang Chen.

**Investigation:** Qiang Chen.

**Methodology:** Lang Chen.

**Supervision:** Qiang Shao.

**Validation:** Qiang Chen.

**Visualization:** Lang Chen.

**Writing – original draft:** Qiang Chen.

**Writing – review & editing:** Qiang Shao.
